# Effects of COVID-19 pandemic–associated reduction in respiratory infections on infantile asthma development

**DOI:** 10.1016/j.jacig.2024.100256

**Published:** 2024-04-11

**Authors:** Chinami Kaiga, Manabu Miyamoto, Takashi Matsushita, Yu Kuramochi, Hiromi Tadaki, Mayumi Enseki, Kota Hirai, Shigemi Yoshihara, Hiroyuki Furuya, Fumio Niimura, Masahiko Kato, Hiroyuki Mochizuki

**Affiliations:** a Department of Pediatrics, Tokai University Hachioji Hospital, Tokyo, Japan; b Department of Pediatrics, Tokai University School of Medicine, Isehara, Japan; c Department of Pediatrics, Dokkyo Medical University, Mibu, Japan; d Tokyo Metropolitan Children’s Medical Center, Tokyo, Japan; e Division of Pediatrics, National Hospital Organization Yokohama Medical Center, Yokohama, Japan; f Department of Basic Clinical Science and Public Health, Tokai University School of Medicine, Isehara, Japan

**Keywords:** Asthma, ATS-DLD questionnaire, COVID-19, infants, RSV, respiratory tract infection

## Abstract

**Background:**

It is speculated that the coronavirus disease 2019 (COVID-19) pandemic–associated reduction in the prevalence of respiratory tract infections has influenced the incidence of asthma in young children.

**Objectives:**

We investigated an association between the reduction in viral infections and the reduction in asthma in young children.

**Methods:**

The subjects were infants born in the early stages of the COVID-19 pandemic in Japan, which began in February 2020. A questionnaire survey related to asthma and allergy was conducted at 18 months and 3 years of age. These results were compared to those of age-matched infants during the nonpandemic period.

**Results:**

There were no epidemics of viral infectious diseases until the target child was 18 months old. At 18 months, the incidence of asthma/asthmatic bronchitis diagnosed by physicians in pandemic children was significantly lower than that in nonpandemic children. In 3-year-olds, no marked difference was observed between nonpandemic infants and pandemic children, except for an increase in respiratory syncytial virus infection in pandemic children. In a comparative study of the same children at ages 18 months and 3 years, an increased prevalence of asthma/asthmatic bronchitis was observed in pandemic children. Furthermore, the incidence of asthma after respiratory syncytial virus infection in pandemic infants was significantly lower than that in nonpandemic children.

**Conclusion:**

The COVID-19 pandemic–associated reduction in respiratory tract infections may have reduced the incidence of asthma in early childhood, and respiratory syncytial virus infection after 18 months of age had little effect on the onset of asthma. These results indicate the importance of preventing respiratory tract infections in early infancy.

Since the beginning of 2020, coronavirus disease 2019 (COVID-19) infection has spread worldwide, resulting in the temporary declaration of a state of emergency by the Japanese government. For approximately 3 years, from February 2020 to April 2023, restrictions were imposed on not only going out and traveling, but also commuting to school and work. As a result, the incidence of viral infections overall decreased.^1^^,^^2^ In Japan, the onset of COVID-19 and other viral respiratory tract infections in infants has been strongly suppressed for some time.^3^

Two factors, atopic predisposition and respiratory tract infection, have been implicated in the pathogenesis of childhood asthma.^4^ Especially in infants, respiratory tract viral infections, including respiratory syncytial virus (RSV) and rhinovirus, are presumed to be important factors related to the onset of asthma in childhood.^5^^,^^6^ Because the long-term decrease in the prevalence of respiratory tract infections related to COVID-19 was considered to be a special environment—one in which the relationship between the development of asthma and respiratory tract viral infections could be evaluated—we planned a prospective observational study on asthma development in infants from June 1, 2021, using a questionnaire survey previously conducted in 2014.^7^ The purpose of this study was to confirm that the long-term suppression of viral respiratory tract infections through the strict infection control measures implemented during the COVID-19 pandemic suppressed the onset of asthma in children.

However, as an unexpected event, in May 2021, about 18 months after the start of the COVID-19 pandemic and when we had just started disseminating the first questionnaire survey on the subjects of this study (18-month-olds), an RSV epidemic occurred worldwide,^8^^,^^9^ including record numbers of cases in Japan,^10^ which led to an additional discussion on RSV infection and asthma development in young children in this report.

## Methods

### Study protocol

The reduction in respiratory tract viral infections was the focus of this study. The Japanese government’s request to close elementary and junior high schools went into effect on March 2, 2020,^3^ so we therefore defined the period before March 1, 2020, as the COVID-19 nonpandemic period. The period from March 2, 2020, until April 30, 2023, when strict infection control measures were lifted by the Japanese government, was defined as the COVID-19 pandemic period.

This study was reviewed and approved by the institutional review board of Tokai University School of Medicine (approval 20R324, March 17, 2021).

### Confirmation of epidemic of viral infection

Our facilities are located on Kanto Plain, the largest plain in Japan, and include large cities such as Tokyo and Yokohama. Yokohama City, which is located almost in the center of this region, has reported the number of patients by disease per sentinel on a weekly basis.^10^ Yokohama City’s detailed surveillance report was thus used as a reference for trends in infectious diseases in this study.

### Subjects

The pediatric departments in the 4 facilities in Tokyo, Kanagawa prefecture, and Tochigi prefecture were those of Tokai University Hospital, Tokai University Hachioji Hospital, Dokkyo Medical University Hospital, and National Hospital Organization Yokohama Medical Center. Children born between December 1, 2019, and April 30, 2020, were defined as pandemic children. The inclusion criteria were that the children had undergone the 18-month-old infant health checkup at our facility between June 1 and September 31, 2021, and their parents gave their consent for their children’s information to be included in this research. Children with neonatal respiratory diseases, cardiac diseases, neurologic diseases, or preexisting COVID-19 infection at entry were excluded.

Children born in 2014 were defined as nonpandemic children. Data of 18-month-olds from May 1 to September 31, 2015, and 3-year-olds from November 1, 2017, to March 31, 2018, were compared to the data of the pandemic children.

Furthermore, we planned to conduct long-term observations to compare changes over time in pandemic and nonpandemic children. To this end, we conducted the aforementioned questionnaire survey twice, at 18 months and 3 years of age, in the same children during the pandemic and nonpandemic periods.

### Questionnaire survey

Using the methods of previous studies,^7^^,^^11^^,^^12^ a questionnaire survey was conducted on the same subjects at 18 months and 3 years of age. Using the American Thoracic Society–Division of Lung Diseases (ATS-DLD) questionnaire survey form (Japanese version; see Table E1 in this article’s Online Repository at www.jaci-global.org),^7^ the first questionnaire survey was conducted after obtaining informed consent from parents at the 18-month-old infant health checkup. The questionnaire included questions on family history, medical history (wheezing, asthma), family smoking, domestic pets, and air pollution. For COVID-19 pandemic children, an additional question about the history of COVID-19 was added. For the questionnaire survey at 3 years old, we sent the questionnaire form in a sealed letter to the homes of the guardians of all participants when participants were 3 years 0 weeks old and asked that the answers be returned to us.

The study protocol was approved by the institutional review board of Tokai University Hospital (approval 20R324, March 17, 2021). We informed participants’ legal guardians about the study, and written informed consent was obtained. For data related to the previous study, opt out was conducted at our outpatient clinic from May 1 to August 15, 2023.

### Statistical analyses

Statistical analyses were conducted by SPSS 22 for Windows (IBM, Armonk, NY). The parameters were compared by Mann-Whitney *U* test. The Pearson chi-square test was used to test for independence. For paired data, *P* values were calculated by McNemar test. Statistical significance was set at *P* < .05.

## Results

### Subjects

A total of 223 children (male:female 117:106) who were 18 months old during the COVID-19 pandemic were enrolled onto this study. A questionnaire survey was conducted on all participants at 3 years 0 weeks old, and 168 children (male:female 91:77) completed the questionnaire survey, resulting in a response rate of 75.3% (Table I). Although the differences were small, statistically significant differences in age and height were observed between the nonpandemic children (mean age, 18 months 3 days and 36 months 21 days) and the pandemic children (18 months 14 days and 37 months 3 days) (Table I).

**Table I:** Characteristics of subjects

Age	Characteristic	Nonpandemic infants	Pandemic infants	*P*
Total, 18 months	No. of subjects	81	223	
	Age (months)	18 (17, 20)	18 (17, 21)	<.001∗
	Sex (male:female)	40:41	117:106	.635
	Height (cm)	79.1 (72.0, 86.4)	80.0 (73.0, 90.0)	.008∗
	Weight (kg)	10.4 (8.2, 15.5)	10.0 (8.0, 13.5)	.964
Total, 3 years	No. of subjects	257	168	
	Age (months)	36 (35, 42)	37 (36, 42)	<.001∗
	Sex (male:female)	132:125	89:79	.656
	Height (cm)	92.0 (84.0, 101.4)	93.0 (84.0, 105.0)	.308
	Weight (kg)	13.8 (10.0, 19.4)	14.0 (10.7, 18.0)	.932
Measured twice in same subject, 18 months	No. of subjects	69	168	
	Age (months)	18 (17, 20)	18 (17, 21)	.012∗
	Sex (male:female)	33:36	91:77	.376
	Height (cm)	79.3 (72.0, 86.4)	80.0 (73.0, 90.0)	.016∗
	Weight (kg)	10.4 (8.2, 15.5)	10.2 (8.0, 13.3)	.331
Measured twice in same subject, 3 years	No. of subjects	69	168	
	Age (months)	36 (35, 39)	37 (36, 42)	<.001∗
	Sex (male:female)	33:36	91:77	.376
	Height (cm)	92.0 (84.0, 101.4)	93.0 (84.0, 105.0)	.009∗
	Weight (kg)	13.6 (10.3, 19.4)	14.0 (10.7, 18.0)	.411

Information is presented as median (minimum, maximum). *P* values were calculated by Mann-Whitney *U* test.

∗ Statistically significant.

A total of 81 (male:female 40:41, data not published) and 257 (male:female 132:125; some of these data were used for a previous lung sound analysis study^7^) young children were included as nonpandemic 18-month-olds and 3-year-olds, respectively. A questionnaire survey was conducted on all 81 participants at 3 years 0 weeks old, and 69 infants had survey information completed, resulting in a response rate of 85.2% (Table I).

### Confirmation of epidemics of viral infections

On the basis of reports on the number of patients by disease for all ages per sentinel in Yokohama City, trends in RSV, influenza virus, and hand, foot, and mouth disease are shown in Fig E1 in the Online Repository at www.jaci-global.org.^10^ In the 18-month period from March 2020, there were no epidemics of respiratory infections. However, from May to July 2021, the Kanto region, including Tokyo, recorded the largest RSV infection to date (Fig E1, *arrowhead*). In summer 2022, an RSV epidemic was also observed, and these trends were confirmed throughout Japan.^3^

In contrast, influenza virus and hand, foot, and mouth disease epidemics have declined sharply since March 2020, and this trend has continued for more than 2 years.^3^ A report from Yokohama City also showed a sharp decrease in the number of people infected with pharyngoconjunctival fever and herpangina during the same period (data not shown). Among pandemic children, the prevalence of COVID-19 was 0.9% in 18-month-olds and 41.1% in 3-year-olds.

### Comparison of questionnaire results in 18-month-olds and 3-year-olds

Among 18-month-olds, rate of physician-diagnosed asthma/asthmatic bronchitis was significantly lower in pandemic children than in nonpandemic children, and the incidence of RSV infection in pandemic children was higher than that in nonpandemic children as a result of the prevalence of RSV at the start of this study (Table II). However, aside from details of domestic pet ownership, there were no marked differences between the 2 groups, including in the incidence of wheezing, recurrent wheezing, atopic disease, family history, and exposure to passive smoking.

**Table II:** Comparison of questionnaire results between non–COVID-19 and COVID-19 children

Age	Question or group	No. (%) with question positive for:		Comparison, OR (95% CI)	*P*
		Nonpandemic infants	Pandemic infants		
18 months	No. of subjects	81	223		
	Q2. History of wheezing	12 (14.8)	24 (10.8)	1.44 (0.69-3.38)	.334
	Q3. Wheezing with cold	27 (33.3)	67 (30.0)	1.16 (0.68-2.01)	.584
	Q4. Recurrent wheezing	8 (9.9)	19 (8.5)	0.85 (0.38-2.03)	.714
	Q5. Dyspnea	7 (8.6)	7 (3.1)	2.92 (1.00-8.60)	.496
	Q7. Asthma/asthmatic bronchitis	16 (19.8)	12 (5.4)	3.67 (1.62-8.33)	<.001∗
	Q8. RSV infection	11 (13.6)	57 (26.5)	0.46 (0.23-0.93)	.027∗
	Q9. Hospitalization	5 (6.2)	5 (2.2)	3.49 (1.03-11.76)	.118
	Q10. Allergy	7 (8.6)	22 (9.9)	0.86 (0.35-2.11)	.749
	Q11. Atopic dermatitis	8 (9.9)	21 (9.4)	1.05 (0.45-2.48)	.904
	Q12. Family history of allergy	58 (71.6)	164 (73.5)	0.91 (0.51-1.60)	.737
	Q13. Passive smoking	24 (29.6)	73 (32.7)	1.34 (0.79-2.27)	.237
	Q14. Domestic pets	30 (37.0)	38 (17.0)	2.86 (1.62-5.07)	<.001∗
	Q15. Air pollution	40 (49.4)	124 (55.6)	0.78 (0.47-1.30)	.337
	Wheezing group	31 (38.3)	72 (32.3)	1.17 (0.69-2.00)	.402
	Atopy group	13 (16.0)	36 (16.1)	0.99 (0.50-1.98)	.984
3 years	No. of subjects	257	168		
	Q2. History of wheezing	23 (8.8)	12 (7.1)	1.16 (0.56-2.43)	.691
	Q3. Wheezing with cold	75 (28.8)	47 (28.0)	1.02 (0.66-1.57)	.924
	Q4. Recurrent wheezing	36 (15.1)	20 (11.9)	0.76 (0.42-1.36)	.354
	Q5. Dyspnea	12 (4.6)	6 (3.6)	1.09 (0.39-3.07)	.710
	Q7. Asthma/asthmatic bronchitis	37 (14.2)	15 (8.9)	1.72 (0.91-3.24)	.093
	Q8. RSV infection	43 (16.5)	68 (40.5)	0.30 (0.19-0.46)	<.001∗
	Q9. Hospitalization	19 (7.3)	5 (3.0)	2.75 (1.00-7.48)	.063
	Q10. Allergy	41 (15.8)	33 (19.6)	0.78 (0.47-1.29)	.327
	Q11. Atopic dermatitis	30 (11.2)	22 (13.1)	0.88 (0.49-1.58)	.662
	Q12. Family history of allergy	61 (75.4)	141 (83.9)	0.65 (0.39-1.06)	.085
	Q13. Passive smoking	78 (30.0)	45 (26.8)	1.19 (0.77-1.84)	.429
	Q14. Domestic pets	56 (21.5)	32 (19.0)	1.05 (0.65-1.71)	.832
	Q15. Air pollution	132 (50.8)	89 (53.0)	0.92 (0.62-1.35)	.656
	Wheezing group	76 (29.2)	45 (26.8)	1.11 (0.72-1.71)	.576
	Atopy group	55 (21.2)	47 (28.0)	0.70 (0.45-1.10)	.121

Wheezing group comprised children with positive responses for wheezing-related items (question [Q] 2 or Q3); atopy group, children with positive responses for atopy-related items (Q10 or Q11). *P* values were calculated by Mann-Whitney *U* test.

∗ Statistically significant.

Among 3-year-olds, except for RSV infection, there were no marked differences in parameters between pandemic and nonpandemic children.

### Comparison of questionnaire results for same children at ages 18 months and 3 years

Among nonpandemic children, no significant differences were observed between ages 18 months and 3 years (Table III). However, among pandemic children, increases in atopic disease, family history of allergy, significant changes in the prevalence of asthma/asthmatic bronchitis, recurrent wheezing, and prevalence of RSV were observed from ages 18 months to 3 years (Table III).

**Table III:** Comparison of questionnaire results between same paired-sample children at ages 18 months and 3 years

Pandemic status	Characteristic	No. (%) with question positive at:		*P*
		18 months	3 years	
Nonpandemic (n = 69)	Q2. History of wheezing	8 (11.6)	7 (10.1)	1.000
	Q3. Wheezing with cold	21 (30.4)	24 (34.8)	1.000
	Q4. Recurrent wheezing	6 (8.7)	9 (13.0)	.250
	Q5. Dyspnea	4 (5.8)	4 (5.8)	.500
	Q7. Asthma/asthmatic bronchitis	13 (18.8)	9 (13.0)	.500
	Q8. RSV infection	8 (11.6)	8 (11.6)	1.000
	Q9. Hospitalization	3 (4.3)	3 (4.3)	1.000
	Q10. Allergy	6 (8.7)	10 (14.5)	.125
	Q11. Atopic dermatitis	6 (8.7)	6 (8.7)	1.000
	Q12. Family history of allergy	48 (69.6)	47 (68.1)	1.000
	Q13. Passive smoking	22 (31.9)	24 (38.4)	1.000
	Q14. Domestic pets	26 (37.9)	28 (40.6)	.500
	Q15. Air pollution	36 (52.2)	32 (46.3)	1.000
	Wheezing group	26 (37.7)	24 (34.8)	.500
	Atopy group	10 (14.4)	14 (20.3)	.125
Pandemic (n = 168)	Q2. History of wheezing	14 (8.3)	12 (7.1)	.607
	Q3. Wheezing with cold	48 (28.6)	47 (28.0)	1.000
	Q4. Recurrent wheezing	9 (5.4)	20 (11.9)	.008∗
	Q5. Dyspnea	3 (1.8)	6 (3.6)	.625
	Q7. Asthma/asthmatic bronchitis	8 (4.8)	15 (8.9)	.016∗
	Q8. RSV infection	43 (25.6)	68 (40.5)	<.001∗
	Q9. Hospitalization	4 (2.4)	5 (3.0)	1.000
	Q10. Allergy	17 (10.1)	33 (19.6)	.001∗
	Q11. Atopic dermatitis	16 (9.5)	22 (13.1)	.210
	Q12. Family history of allergy	123 (73.2)	141 (83.9)	<.001∗
	Q13. Passive smoking	52 (31.0)	45 (26.8)	.065
	Q14. Domestic pets	30 (17.9)	32 (19.0)	.424
	Q15. Air pollution	87 (51.8)	89 (53.0)	.875
	Wheezing group	52 (31.0)	45 (26.8)	.311
	Atopy group	28 (16.7)	47 (28.0)	<.001∗

Wheezing group comprised children with positive responses for wheezing-related items (question [Q] 2 or Q3); atopy group, children with positive responses for atopy-related items (Q10 or Q11). *P* values were calculated by McNemar test.

∗ Statistically significant.

### Association of RSV infection and asthma onset

Regarding the relationship between RSV infection and other factors in nonallergic children, children with RSV infection at all ages were more likely to develop recurrent wheezing, to develop asthma/asthmatic bronchitis, and to be hospitalized for respiratory tract infections than children without RSV infection (see Table E2 in the Online Repository at www.jaci-global.org). Therefore, we focused on the incidence of asthma with RSV infection (Table IV), and the onset of asthma after RSV infection was compared between the 2 groups.

**Table IV:** Effect of RSV infection on incidence of asthma or asthmatic bronchitis

Positive RSV infection at:	Characteristic	Nonpandemic children	Pandemic children	Total no. of children
18 months (n = 68)∗	Asthma	7	6	13
	No asthma	4	51	55
	Total	11	57	68
3 years (n = 111)†	Asthma	15	11	26
	No asthma	28	57	85
	Total	43	68	111

∗ Odds ratio (95% confidence interval) = 14.88 (3.35-66.10), *P* < .001.

† Odds ratio (95% confidence interval) = 2.78 (1.13-6.83), *P* = .023.

Among 18-month-olds during the nonpandemic period (n = 81), 11 had RSV infection and 7 (63.6%) had asthma (Table IV). Among 18-month-olds during the pandemic period (n = 223), 57 were infected with RSV, and 6 (10.5%) developed asthma. The prevalence of asthma after RSV infection in pandemic infants was significantly lower than that in nonpandemic children (*P* < .001). Among 3-year-olds during the nonpandemic period (n = 257), 43 children had RSV infection and 15 (34.9%) had asthma. Among 3-year-old children during the pandemic period (n=168), 68 were infected with RSV, and 11 (16.2%) developed asthma. The prevalence of asthma after RSV infection in pandemic children was significantly lower than that in nonpandemic children (*P* = .023).

## Discussion

This large-scale prospective study shows that the marked decline in the incidence of respiratory tract infections during the COVID-19 pandemic period reduced the incidence of asthma among children. It was also confirmed that RSV infection after 18 months of age has little effect on the onset of childhood asthma. These results are valuable evidence showing the relationship between the onset of childhood asthma and respiratory tract infections in early childhood.

During the nearly 3-year-long COVID-19 pandemic, unprecedented attempts have been made worldwide to prevent infection, and both children and adults have been encouraged to wear masks, wash their hands, and keep their distance from others.^1^^,^^13^ In Japan, children in kindergartens and elementary and junior high schools have been forced to limit class hours and change class formats over a long period of time.^14^ Thus, it is speculated that these actions significantly reduced the prevalence of respiratory tract infections in children during the pandemic. In fact, with reference to local government data in our region showing weekly incidences of viral infections, during the period from birth to 18 months of age, the number of infections caused by RSV, influenza virus, and coxsackie virus was extremely low among the infants who were the subject of this study.^10^

However, there has been much debate regarding the development of asthma in infants. There are many reports on the onset of asthma related to atopic predisposition and viral infections in infants,^15^^,^^16^ and both are considered important risk factors for the onset of asthma. As a trial to definitively demonstrate the development of viral infections and recurrent wheezing/asthma, studies have been conducted concerning the administration of palivizumab, a therapeutic agent for anti-RSV antibodies, which shows a suppression on the development of recurrent wheezing/asthma.^17^^,^^18^

Therefore, a reduction in respiratory virus infections may reduce the development of childhood asthma. To date, few prospective studies have investigated how the reduction in respiratory viral infections due to the COVID-19 pandemic affects the development of asthma, and even fewer have considered the long-term effects.^9^ We believe that we can clearly demonstrate the association between a reduction in viral infections and recurrent wheezing/asthma in children.

In the present study, the incidence of physician-diagnosed asthma/asthmatic bronchitis was significantly reduced in 18-month-olds who were born and grew up during the pandemic. In 3-year-olds, there was also a trend toward a reduced incidence of physician-diagnosed asthma/asthmatic bronchitis, although no statistically significant difference was observed (*P* = .093). There is no doubt that atopic constitution affects the development of asthma in infants with age.^16^ Furthermore, given the increasing prevalence of respiratory tract viral infections, we believe that the effects of the COVID-19 pandemic on reducing the incidence of asthma are likely to be short-lived and limited to children with infection-related asthma.^19^ These results indicate that the mechanisms underlying the development of the 2 representative phenotypes of childhood asthma, infectious asthma and atopic asthma, maintain some degree of independence.^17^, ^18^, ^19^ Because the clinical course itself differs between these 2 phenotypes, long-term management methods will likely differ as well. It seems important to proceed with medical treatment in the future while recognizing this point.

An analysis based on survey results from participants found that rates of home smoking and air pollution, which are considered risk factors for developing asthma, did not change significantly between the pandemic and nonpandemic periods.^20^, ^21^, ^22^ This suggests that the decrease in the prevalence of respiratory tract infections was directly associated with a subsequent reduction in the incidence of asthma. Although a decrease in domestic pet-keeping rates was observed in 18-month-olds, no marked difference was observed in 3-year-olds, suggesting that there was no significant effect.

In the comparison of the same subject at 18 months and 3 years of age, the prevalence of wheezing, asthma/asthmatic bronchitis, and allergic diseases increased over time as a natural trend. However, the change in the pandemic period was greater than that in the nonpandemic period (Table III). Interestingly, the onset of allergic diseases was suppressed in parallel with the suppression of infectious diseases in infants up to 18 months old during the pandemic period, likely due to the effects of restrictions on going out or the encouragement to wear masks. At present, the magnitude of the impact of COVID-19 on the lives of these children has not yet been affirmed, and no correct conclusion can be assumed. However, at 3 years old, a sudden increase in allergic diseases appeared to have little effect on the development of recurrent wheezing or asthma (Table E2).

Interestingly, there was no RSV epidemic in our area from December 2020 to May 2021, so for most pandemic children, the first RSV infection occurred after 18 months of age. Furthermore, the RSV epidemic in May-July 2021 was the largest ever observed in the Kanto region.^10^ Many previous studies have reported that children infected with RSV develop asthma at a certain rate.^16^^,^^23^^,^^24^ In this report, a comparison of the incidence of asthma after RSV infection between nonpandemic and pandemic children showed that the incidence of asthma in pandemic children was markedly lower than that in nonpandemic children (*P* < .001, Table IV). It is speculated that damage to the respiratory tract mucosa caused by RSV infection remains more pronounced in immature children than in older children and is more likely to lead to childhood asthma.^25^^,^^26^ In contrast, children over 18 months of age appear to have effective protection against RSV infections. This result clearly indicates the need for prevention of RSV in early infancy. Based on previous reports,^17^^,^^18^ these results seem important not only concerning the use of biological products that have a clear preventive effect against RSV infection, but also with regard to the application of vaccines against RSV that may be used clinically in the near future. We think that this is an important finding to discuss.

Although COVID-19 affected 41.1% of the pandemic children at 3 years old in this study, previous reports suggested that COVID-19 had little effect on the onset of asthma.^27^ Among respiratory tract viruses, rhinovirus has attracted attention as an onset factor for childhood asthma.^28^ Unfortunately, we were unable to provide data on the increase or decrease in rhinovirus in our district at this time. According to a previous report on the incidence of rhinovirus in Japan,^3^ the number of rhinovirus-infected people clearly decreased from February 2020, and this decline continued until at least summer 2021. This suggests that the impact of rhinovirus infection on childhood asthma would have been negligible until the outbreak of RSV in summer 2021.

The impact of COVID-19 will likely last for a long time, and the pandemic has had a tangible and intangible impact on the lives of all citizens, including mental aspects,^29^ which must be taken into consideration. Our study found no significant difference in the smoking rate at home, even during the pandemic period, whereas the pet ownership rate decreased at age 18 months and recovered at age 3 years. Considering previous reports of the relationship between pet ownership and asthma onset, the impact of these changes may not have been large.^30^ However, it is conceivable that unexpected changes were related to the onset of asthma throughout the lives of the children.

The COVID-19 pandemic had a clear effect on the frequency and system of health checkups performed by the local government, as well as on the questionnaire responses of parents in this study. Compared to the nonpandemic period, delays in receiving health checkups and responding to questionnaires were conspicuous during the pandemic period. However, because the discrepancy was only 2 weeks, it was thought that this delay would not have had a significant impact on this study.

One limitation of this study is that we conducted a self-reported survey among parents using a questionnaire. Disadvantages associated with self-reporting include the possibility that survey subjects may not accurately remember their child’s medical history, which must be considered when evaluating study results. Another limitation is that the ATS-DLD questionnaire, Q7, asked about both asthma and asthmatic bronchitis, although they were evaluated by doctors. However, it is an evaluation at young age, when it is difficult to clearly diagnose asthma.^31^ This survey was conducted in children up to 3 years old, and the lack of clarity in the diagnosis of asthma development compared to studies in children 5 years old or older was considered to be a limitation.

Furthermore, there was no marked difference in the occurrence of wheezing between the pandemic and nonpandemic children. However, in previous studies, it was difficult for mothers to distinguish between wheezing originating from the upper versus the lower respiratory tract.^32^ The pandemic group also experienced epidemics of RSV infections at age 18 months, which may explain the lack of a marked difference in the results regarding the presence or absence of wheezing between the 2 groups. In addition, we did not investigate changes in smoking habits of family and in outdoor air pollution during the COVID-19 pandemic, including their relationship to indoor pollution. A further limitation of this study is that it is based on a limited number of survey responses.

Consequently, our results suggest that the incidence of asthma in children may have been suppressed during the 18 months when viral infections were significantly reduced by the COVID-19 pandemic in Japan. Our data are valuable in indicating a relationship between the onset of asthma and respiratory tract infections during infancy and young childhood. Furthermore, RSV infection after 18 months of age may have little effect on the development of childhood asthma. The unique circumstances surrounding this investigation cannot be repeated, and the results are of great significance in preventing the onset and exacerbation of childhood asthma.

## Disclosure statement

Supported by the 10.13039/100014423Environmental Restoration and Conservation Agency, Japan in fiscal years 2019 to 2021.

Disclosure of potential conflict of interest: The authors declare that they have no relevant conflicts of interest.

## Supplementary data

Supplementary Tables E1 and E2

Supplementary Fig E1
