# Supplementary material for: Effects of COVID-19 pandemic–associated reduction in respiratory infections on infantile asthma development
Source: J Allergy Clin Immunol Glob. 2024 Apr 11;3(3):100256. doi: 10.1016/j.jacig.2024.100256 (PMC11090864; doi:10.1016/j.jacig.2024.100256)
Supplement: Supplementary Tables E1 and E2 [file mmc1.docx]

Table E1. Original questionnaire (translated to English)

Child’name　　　　　　　　　 Address 　Sex (1, Male　2, Female)

Birthday ( __ /__/20___), Age ___ years___months, Height ____cm, Weight ____kg

Q1. Has your child recently caught an acute respiratory infection?

1. He/She has not had an acute respiratory infection for a week.

2. He/She recovered from an acute respiratory infection ( ) days ago.

3. He/She has an acute respiratory infection now.

Q2. When your child breathes, have you heard the sound of wheezing or whistling?

(1, Yes　2, No)

Q3. When your child has had a cold, have you heard a wheezy or whistling sound?

(1, Yes　2, No)

Q4. How many times has your child’s chest sounded wheezy?

(1, 0 times, 2, 1-2 times, 3, 3-6 times, 4, 7-12 times, 5, More than 13 times)

Q5. Has your child suffered from attacks characterized by difficulty breathing with wheezing

or whistling?

(1, Yes　2, No)

Q6. If yes, how many such attacks has your child had?

(1, 0 times, 2, 1-2 times, 3, 3-6 times, 4, 7-12 times, 5, More than13 times)

Q7. Has your child been diagnosed with bronchial asthma or asthmatic bronchitis by

a physician? (1, Yes　2, No)

Q8. Has your child been diagnosed with an RS virus-induced respiratory infection?

(1, Yes　2, No)

Q9. Has your child been hospitalized because of bronchial asthma, bronchitis or pneumonia?

(1, Yes　2, No)

Q10. Does your child have any allergies? (1, Yes　2, No)

If yes and a blood test was performed, please select all that were positive.

(1.Mites 2.House dust 3.Cedar Pollen 4.Cat dander 5.Egg white 6.Milk 7.Others [ ])

Q11. Has your child been diagnosed with atopic dermatitis by a physician? (1, Yes　2, No)

Q12. Do your child's family have any of the allergic diseases described below? Please connect

the corresponding upper and lower words with a line.

[ Asthma, Allergic rhinitis (hay fever), Atopic dermatitis, Others]

[Father, Mother, Siblings, Grandparents]

Q13. Is there anyone who smokes in your house? Please circle all that apply.

(1, Father　2, Mother　3, Others　4, None)

Q14. What kind of domestic pets do you keep?　 　(1, Dog 2, Cat　3, Others　4, None)

Q15. Is there a road with heavy traffic near the house? 　　　　 　(1, Yes　2, No)

Table E2. Effect of RSV infection on asthma-related questions in non-allergic infants

1. 18 months Non-pandemic infants (n=68) Pandemic infants (n=187)

RSV infection Positive RSV infection Positive

OR (95% CI) P^a^ OR (95% CI) P

Q2, History of wheezing 11.0 (2.09-57.90) **0.001** 2.70 (1.03- 7.09) **0.038**

Q3, Wheezing with cold 2.75 (0.61-12.32) 0.177 6.04 (2.99-12.20) **<0.001**

Q4, Recurrent wheezing 0.14 (0.07- 6.26) 0.698 0.38 (0.14- 1.05) 0.055

Q5, Dyspnea - **<0.001** 4.19 (0.68-25.83) 0.097

Q7, Asthma/asthmatic bronchitis 9.00 (1.78-45.58) **0.003** 8.93 (1.74-45.85) **0.002**

Wheezing group^b^　　　　　　　3.89 (0.84-18.03) 0.070 5.51 (2.75-11.05) **<0.001**

1. 3 years Non-pandemic infants (n=202) Pandemic infants (n=121)

RSV infection Positive RSV infection Positive

OR (95% CI) P OR (95% CI) P

Q2, History of wheezing 3.29 (1.03-10.57) **0.036** - **0.022**

Q3, Wheezing with cold 2.80 (1.27- 6.14) **0.009** 2.40 (1.03- 5.58)  **0.040**

Q4, Recurrent wheezing 0.22 (0.09- 0.58) **0.001** 0.41 (0.11- 1.49) 0.166

Q5, Dyspnea 4.29 (0.91-20.18) 0.077 -  **0.048**

Q7, Asthma/asthmatic bronchitis 6.33 (2.51-15.92) <**0.001** 10.20 (1.21-85.68) **0.010**

Wheezing positive　　　　　　2.80 (1.27- 6.15) **0.009** 2.98 (1.24- 7.19) **0.013**

a: P values were calculated by Mann-Whitney U-test. b: Wheezing group; infants with positive responses for wheezing-related items (Question 2 or 3), c: Atopy group; infants with positive responses for atopy-related items (Question 10 or 11). Bold letters represent values with a significant difference. “-“ means no data, as one of the cells in a 2-by-2 table is 0.
