# Supplementary material for: Effects of COVID-19 pandemic–associated reduction in respiratory infections on infantile asthma development
Source: J Allergy Clin Immunol Glob. 2024 Apr 11;3(3):100256. doi: 10.1016/j.jacig.2024.100256 (PMC11090864; doi:10.1016/j.jacig.2024.100256)

**Figure legend**

**Figure E1: Prevalence of virus infection in Yokohama City from June, 2019 to April, 2023.**

Weekly reports of newly diagnosed cases of respiratory infections in adults and children in Yokohama City; RSV (solid line), influenza virus (thick dashed line) and hand-foot-mouth disease (thin dashed line). Arrow head: peak of RSV infection in summer 2021.

**Figure E1**


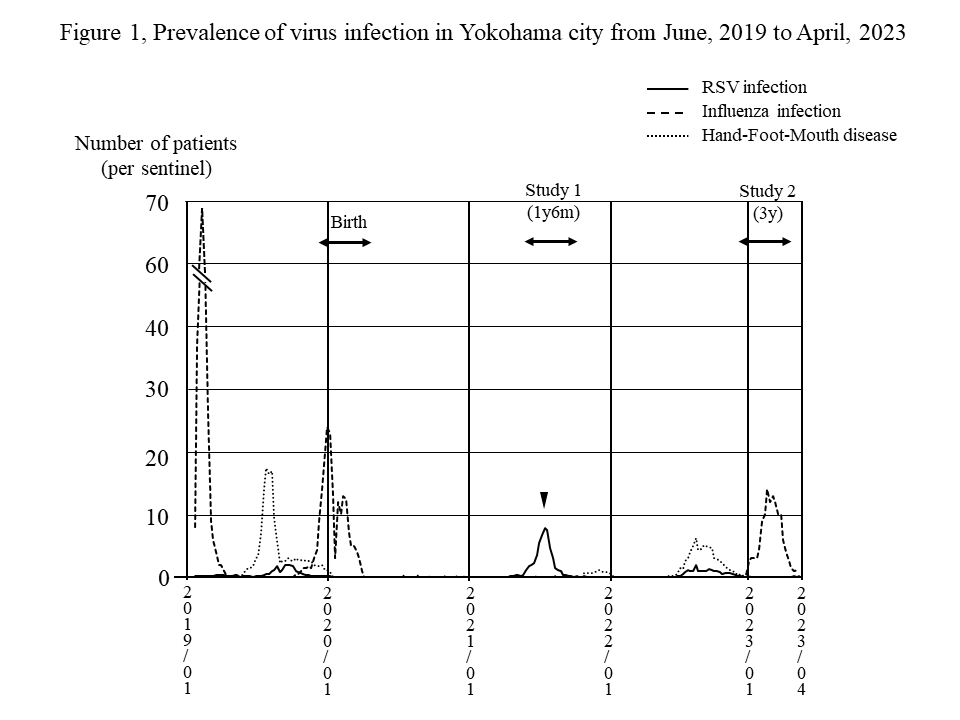

Supplement: Supplementary Fig E1 [file mmc2.docx]
